# High–Performance Biscrolled Ni–Fe Yarn Battery with Outer Buffer Layer

**DOI:** 10.3390/ijms24021067

**Published:** 2023-01-05

**Authors:** Jin Hyeong Choi, Juwan Kim, Jun Ho Noh, Gyuyoung Lee, Chaewon Yoon, Ui Chan Kim, In Hyeok Jang, Hae Yong Kim, Changsoon Choi

**Affiliations:** 1 Department of Energy and Materials Engineering, Dongguk University, 30 Pildong-ro, 1-gil, Jung-gu, Seoul 04620, Republic of Korea; 2 Department of Advanced Battery Convergence Engineering, Dongguk University, 30 Pildong-ro, 1-gil, Jung-gu, Seoul 04620, Republic of Korea; 3 Research Center, Sillo Incorporation, 30 Pildong-ro, 1-gil, Jung-gu, Seoul 04620, Republic of Korea

**Keywords:** biscrolled yarn, Ni–Fe batteries, high load, durability, carbon nanotubes

## Abstract

The increasing demand for portable and wearable electronics has promoted the development of safe and flexible yarn–based batteries with outstanding electrochemical properties. However, achieving superior energy storage performance with a high active material (AM) load and long cycle life with this device format remains a challenge. In this study, a stable and rechargeable high–performance aqueous Ni–Fe yarn battery was constructed via biscrolling to embed AMs within helical carbon nanotube (CNT) yarn corridors. Owing to the high load of charge storage nanoparticles (NPs; above 97 wt%) and the outer neat CNT layer, the buffered biscrolled Ni–Fe yarn battery demonstrates excellent linear capacity (0.053 mAh/cm) and cycling stability (60.1% retention after 300 charge/discharge cycles) in an aqueous electrolyte. Moreover, our flexible yarn battery exhibits maximum energy/power densities of 422 mWh/cm^3^ and 7535 mW/cm^3^ based on the total volume of the cathode and anode, respectively, which exceed those reported for many flexible Ni–Fe batteries. Thus, biscrolled Ni–Fe yarn batteries are promising candidates for next–generation conformal energy solutions.

## 1. Introduction

Owing to the increasing demand for personal health–monitoring devices, intelligent garments, rollup displays, and implantable medical devices, researchers have made considerable progress in the field of flexible and wearable electronics over the past few years [1,2,3,4,5,6]. Because of a rapidly growing market, researchers are increasingly interested in different kinds of fibrous power sources such as lithium–ion batteries, zinc–ion batteries, primary batteries, and supercapacitors for the development of 1D linear energy storage devices [7,8,9,10,11,12,13,14,15,16]. For example, lightweight, flexible, and wearable yarn–based aqueous batteries with tiny volumes are one of the most fascinating candidates for wearable electronics [17,18,19,20,21,22].

Researchers and engineers have tried many strategies to manufacture yarn–shaped batteries: for example, chemical vapor deposition, hydrothermal synthesis, electrodeposition, and in situ polymerization [23,24,25,26,27,28]. Nevertheless, producing high–performance fibrous batteries with a sufficient mass load and long cycle life remains a challenge [29,30,31]. The biscrolling method, which enables the straightforward incorporation of active materials (AMs) into carbon nanotubes (CNTs), is an eminent choice, as it maximizes the AM load (>90 wt%) without the need for binders and laborious procedures [32,33]. Thus, various AMs can be integrated into flexible CNT–based yarn–structured batteries. The easy control of the diameter and porosity of the yarn provides the AMs with steady access to the electrolyte. More importantly, wearable batteries must be suitable for the direct contact with human skin; they should not explode or ignite. Aqueous Ni–Fe batteries, which are traditional aqueous batteries, are relatively safe and cost–effective and exhibit high energy densities [34,35].

In this study, we built a flexible biscrolled Ni–Fe yarn battery (i.e., “BNF yarn battery”) with a Ni(OH)_2_ cathode, Fe_3_O_4_ anode, and water–based electrolyte. Owing to the high AM content (Ni content: up to 98.5 wt%; Fe content: up to 97.4 wt%), the fabricated BNF yarn battery with 1.1 V voltage provides a linear capacity of 0.053 mAh/cm at 0.1 mA/cm current density in an aqueous electrolyte. Because of the outer neat CNT buffer layer, our BNF yarn battery exhibits 60.1% capacity retention for over 300 cycles with high coulombic efficiency. This results in a high energy density of 422 mWh/cm^3^ and a high power density of 7535 mW/cm^3^, which are better than those of the most previously reported flexible Ni–Fe batteries. The proof–of–concept incorporation of mechanically flexible BNF yarn batteries with these properties into textiles renders them promising candidates for wearable electronics.

## 2. Results

### 2.1. Preparation and Morphology of Biscrolled AM/CNT Yarns

**Figure 1:**
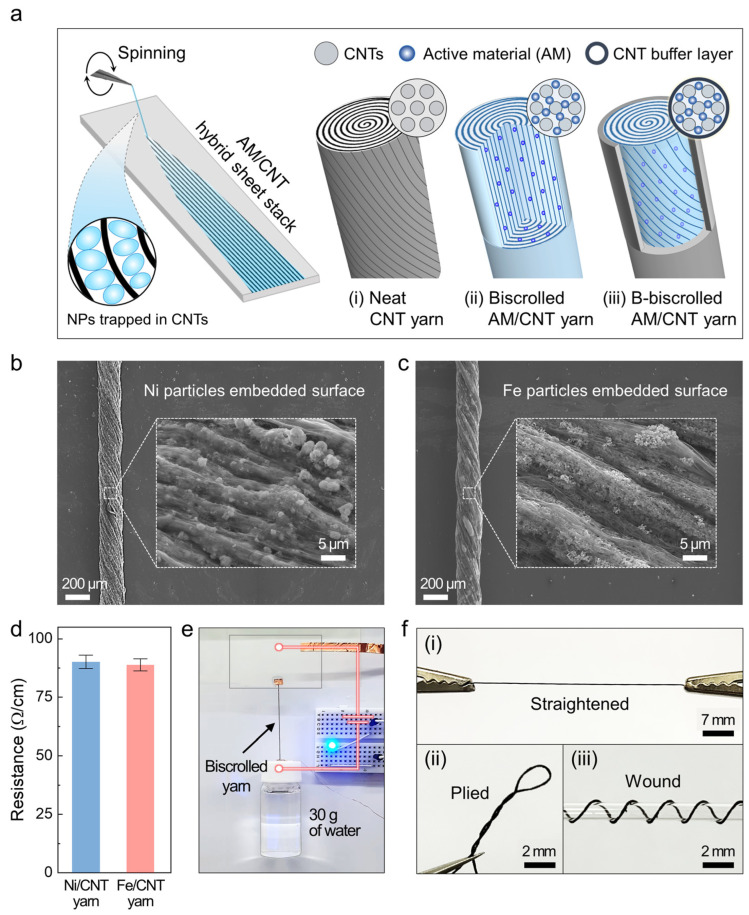
Fabrication of a biscrolled AM/CNT yarn with outer buffer layer. (**a**) Schematic of fabrication of biscrolled AM/CNT yarns. The AM/CNT hybrid sheets were scrolled and wrapped into a neat CNT layer to build a buffered biscrolled AM/CNT yarn (i.e., a b–biscrolled AM/CNT yarn) in which the AM NPs are uniformly distributed within the CNT framework. The SEM images and magnified areas (insets) show the surfaces of (**b**) a 98.5 wt% Ni–embedded cathode yarn and (**c**) a 97.4 wt% Fe–embedded anode yarn. (**d**) Length–normalized resistances of biscrolled Ni/CNT (blue bar) and Fe/CNT (red bar) yarns. (**e**) A biscrolled Ni/CNT yarn bearing a 30 g glass vial filled with water is connected to a power source to power a blue LED. (**f**) Photographs of (**i**) straightened and (**ii**) plied biscrolled Ni/CNT yarns and (**iii**) those wound around a glass tube.

We employed CNT sheets that were drawn from spinnable CNT forests [36] as the host material to form a twist–spun yarn. The guest–free neat CNT yarns (Figure 1(i)) have highly oriented and porous structures (Figure S1); thus, active guest materials can be trapped within the CNT network. We fabricated the biscrolled AM/CNT yarns (Figure 1(ii)) with the typical biscrolling method [33]: an AM/CNT hybrid sheet stack was prepared by drop–casting a dispersion of charge storage nanoparticles (NPs) onto stacked CNT sheets and spinning them at approximately 1200 turns/m. The resulting NP load was tuned by varying the AM concentration that was dispersed in the ethanol. More importantly, the outermost layer of the as–fabricated biscrolled yarn was wrapped into neat CNT sheets to ensure the anchoring of the AMs (Figure S2); that is, the CNT wrapping layer serves as a buffer layer that prevents the AMs from being desorbed during the electrochemical reaction. Thereby, the buffered biscrolled AM/CNT yarns (i.e., the b–biscrolled AM/CNT yarns) are expected to exhibit a high energy storage capacity and excellent cycling durability owing to the maximized weight percentage of the guest without compromising the accessibility of the electrolyte to the guest.

More specifically, 98.5 wt% Ni(OH)_2_ NPs and 97.4 wt% Fe_3_O_4_ NPs were embedded as the AMs in the cathode and anode yarns, respectively. The scanning electron microscopy (SEM) images of the surface and magnified area of the guest NP–loaded biscrolled yarn are shown in Figure 1b,c. Despite the low content of the CNT host, the guest materials are embedded within the CNT scroll galleries. Moreover, a considerable amount of partially aggregated guest material resides on the biscrolled yarn surface, leaving the possibility of a detachment of the AMs. These biscrolled yarns exhibit stable electrical resistance (approximately 90 ohm/cm) irrespective of the AM type, which represents the resistance of the CNT host (Figure 1d). Moreover, a 5 cm long and 200 μm thick biscrolled Ni/CNT yarn was attached to a conductive film and used in an electrical circuit (Figure 1e). The biscrolled yarn provided the blue light–emitting diode (LED) in the circuit with power even when a glass vial filled with water was attached to the yarn (30 g, which is ten thousand times heavier than the yarn); thus, the yarn possesses superior mechanical properties even at a high load of brittle guest NPs. In addition, the as–prepared biscrolled yarn was strong and flexible enough to be (i) straightened, (ii) plied, and (iii) wound around a 2 mm diameter glass tube, as shown in Figure 1f.

### 2.2. Electrochemical Properties of the BNF Yarn Battery

The electrochemical properties of the Ni(OH)_2_ cathode and Fe_3_O_4_ anode were determined with cyclic voltammetry (CV) experiments in the three–electrode configuration with a 6M potassium hydrate (KOH) solution. Figure S3a shows a pair of redox peaks of Ni(OH)_2_ at 0–0.55 V versus Ag/AgCl reference electrode, which correspond to the reversible redox reactions between Ni^2+^ and Ni^3+^ with OH^−^ [37,38]. As shown in Figure S3b, the CV curves of Fe_3_O_4_ display one dominant pair of redox peaks (at approximately −1.1 and −0.7 V) and other small peaks (at approximately −0.8 V), which originate from the redox reaction between Fe^2+^/Fe^3+^ and Fe^0^ with OH^−^. This result agrees well with previously published results for Fe_3_O_4_–based electrodes [39]. Based on these results, BNF yarn–based full cells were fabricated with the Ni(OH)_2_/CNT biscrolled yarn as the cathode and the Fe_3_O_4_/CNT biscrolled yarn as the anode in aqueous electrolyte (Figure S4). The linear capacity versus the mass ratio of Ni/Fe is presented in Figure S5. The optimal Ni(OH)_2_/Fe_3_O_4_ mass ratio is 1.8 for a reasonable and excellent charge balance between the cathode and anode. Figure S6 shows typical CV curves of the BNF yarn battery for different scan rates. The well–defined pair of redox peaks indicates good agreement with the following reaction in the Ni–Fe battery system [40]:

NiO+18Fe3O4+12H2O ↔ NiOOH+38Fe

**Figure 2:**
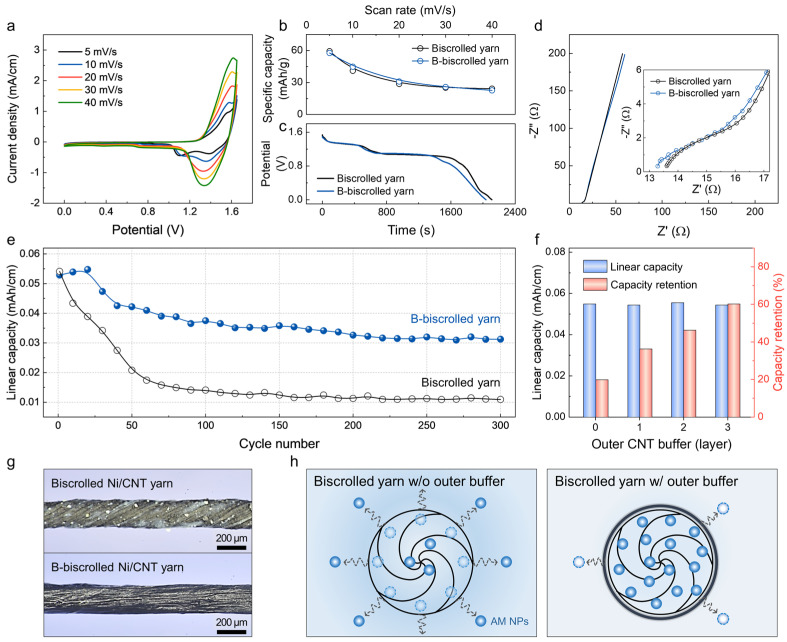
Electochemical characterizations of the BNF yarn batteries. (**a**) CV curves of b—BNF yarn battery in aqueous 6M KOH at different scan rates. (**b**) Average specific capacity as a function of the scan rate, (**c**) galvanostatic discharge curves at 0.1 mA/cm, (**d**) Nyquist plots (from 10 Hz to 100 kHz), and (**e**) cycling stability of BNF and b—BNF yarn batteries at 0.1 mA/cm. Inset in d shows enlarged section of Nyquist plots. (**f**) Dependence of linear capacity and capacity retention on outer CNT buffer layer. (**g**) Optical images show biscrolled Ni/CNT yarn (**top**) and b—biscrolled Ni/CNT yarn (**bottom**). (**h**) Schematics show the expected AM NP desorption process (indicated by arrows) of the biscrolled yarns without (**left**) and with (**right**) an outer CNT buffer layer in an aqueous electrolyte.

The CV curve of the b–BNF yarn battery exhibits a similar trend at the presented scan rates, thereby indicating that the electrolyte sufficiently penetrated the biscrolled yarn and that the embedded AMs could fully participate in the electrochemical reactions with sufficient contact to the electrolyte. Thereby, the BNF and b–BNF yarn batteries provided similar specific capacities (from 22.5 to 59.3 mAh/g based on the total mass of the AMs) and rate capacities at different scan rates (Figure 2b). The specific capacity (C_sp_) was calculated from the CV curves using the following equation:${\;C}_{\mathrm{sp}}(\mathrm{mAh}/g)=\int i/\mathrm{mdt}$, where i is the oxidation or reduction current, dt is the time differential, and m is the total mass of the AMs. In addition, both had similar discharge plateaus and periods (Figure 2c). These results confirm that the neat CNT sheath has no noticeable influence on the capacity of the BNF yarn battery. Evidently, the CNT layer only functions as a buffer layer without participating in the electrochemical reaction of the Ni–Fe battery. The effect of the CNT buffer layer on the electrochemical performance was further investigated with electrochemical impendence spectroscopy (EIS; Figure 2d). Its Nyquist plots (frequency range from 10 Hz to 100 kHz) clearly show that both the equivalent series resistance (i.e., the intersection of the curve at the real part) and the inclined curve in the low–frequency region (i.e., the ion diffusion impedance) of the BNF yarn battery substantially overlapped with those of the b–BNF yarn battery. Furthermore, both batteries revealed the absence of a semicircle in the high–frequency range, thereby indicating the high conductivity of the yarns and the low charge transfer resistance. Notably, the low impedance at 10 Hz (<200 Ω) implies that the ions could easily enter the biscrolled yarn from the aqueous electrolyte even with a CNT buffer layer. More specifically, the series resistance of the b–biscrolled yarn at 100 kHz was 13.3 Ω and, thereby, lower than that of the biscrolled yarn (13.6 Ω; inset of Figure 2d). This slight reduction due to the additional CNT wrapping layer can be explained by the fact that the CNTs provide an enhanced conductive network and promote electron transport.

The electrochemical stability of Ni–Fe batteries is a great concern regarding their widespread commercialization. The cycling performance of our b–BNF yarn batteries was investigated by repeatably charging/discharging them at 0.1 mA/cm. As shown in Figure 2e, the b–BNF yarn battery retained approximately 60.1% of its initial capacity after 300 cycles, whereas the BNF yarn battery showed a short cycle life with only approximately 20.2% capacity retention. The slight increase in the capacity in the initial stage can be attributed to the progressive permeation of the electrolyte with the activation of the AMs [37,41]. The improved durability comes from the protecting neat CNT sheath; the effect is similar to the previously reported buffering behavior of PEDOT shells [42]. Figure 2f shows the linear capacity and capacity retention versus the number of outer CNT buffer layers. It should be noted that the linear capacity of approximately 0.053 mAh/cm remained almost unchanged despite the increase in the number of buffer layers, while the capacity retention rate gradually increased. The buffering effect of the outermost CNT layers was confirmed by taking optical images of the yarns before and after the cycling test. According to Figure 2g, a large amount of exposed Ni particles on the surface of the biscrolled yarn was completely covered by the CNT buffer layer, which resulted in good dissolution tolerance to the electrolyte solution. The biscrolled Ni/CNT yarn without the outer buffer experienced evident Ni particle desorption after 300 cycles, and its surface became smooth (Figure S7, upper image). By contrast, the surface morphology of the b–biscrolled Ni/CNT yarn was well preserved (Figure S7, lower image). Figure 2h presents the predicted dissolution model of the AM NPs embedded in the biscrolled yarns. In the case of the biscrolled yarn without the external buffer, the AM may be desorbed through the yarn surface into the electrolyte because of the aggressive electrochemical reaction. This adverse effect does not occur in the biscrolled yarn with the outer buffer layer, which confines the AM in the yarn.

### 2.3. Performance and Practical Applications

**Figure 3:**
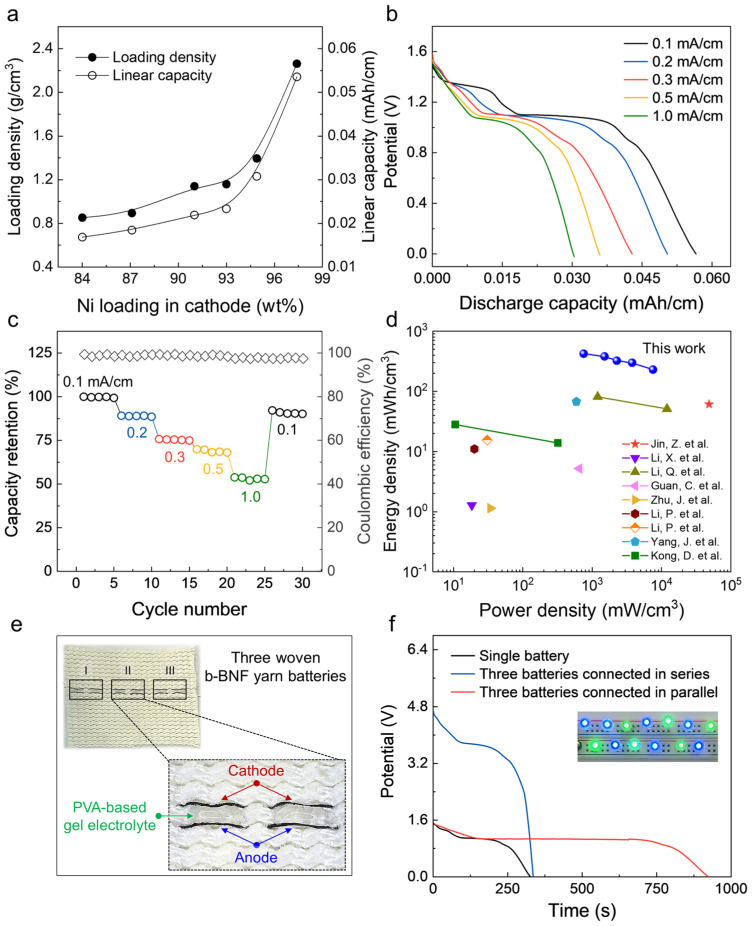
Performances of the b–BNF yarn batteries and demonstration as a practical power source. (**a**) Linear capacity versus Fe load in anode (Ni–to–Fe mass ratio is 1.8:1). (**b**) Galvanostatic discharge curves at different current densities. (**c**) Capacity retention and corresponding coulombic efficiency of b–BNF yarn battery. (**d**) Ragone plot of as–fabricated Ni–Fe battery (based on total volume of the cathode and anode) [45,46,47,48,49,50,51,52,53]. (**e**) Photographs showing three b–BNF yarn batteries woven into a commercial textile. Inset shows that each cell consists of a 3 cm–long Ni(OH)_2_ cathode yarn and a 3 cm–long Fe_3_O_4_ anode yarn with a gel electrolyte (PVA–KOH). (**f**) Galvanostatic discharge curves of single b–BNF yarn battery (black curve) and three batteries connected in series (blue curve) and parallel (red curve) at 1 mA/cm. The inset shows 13 LEDs (seven blue and six green) illuminated by three serial–connected b–BNF yarn batteries.

In this work, the amount of AMs in the yarn was controlled by altering their dispersion concentrations; this resulted in a volumetric loading density and corresponding linear capacity of 2.26 g/cm^3^ and 0.053 mAh/cm, respectively (Figure 3a). This high load can be ascribed to the strong compressive forces generated by the inserted twists [33]. The galvanostatic discharge curves measured at different current densities (from 0.1 to 1 mA/cm) and the corresponding capacity retention rate against the number of charge/discharge cycles are presented in Figure 3b and Figure 3c, respectively. All curves display distinct discharge voltage plateaus at approximately 1.1 V, which are consistent with the CV curves in Figure 2a. This b–BNF yarn battery exhibited a high linear capacity of 0.053 mAh/cm at 0.1 mA/cm current density and 0.031 mAh/cm at 1 mA/cm current density, thereby indicating its good rate capability. In addition, the coulombic efficiency was approximately 100% during rate cycling; hence, the battery is a feasible power supply [40,43,44]. More importantly, when the total volume of the cathode and anode is considered, our b–BNF yarn battery yielded an incredibly high energy density of 422 mWh/cm^3^ at 753 mW/cm^3^ power density. Even at 7535 mW/cm^3^, the energy density was 229 mWh/cm^3^. Thus, the proposed b–BNF yarn battery outperforms most previously reported Ni–Fe batteries (Figure 3d) [45,46,47,48,49,50,51,52,53].

To demonstrate the viability for power source application in wearable electronic devices, the three b–BNF yarn batteries were assembled in series or parallel. Figure 3e exhibits that 3 cm long, three b–BNF yarn batteries can be woven into a commercial textile with a poly(vinyl alcohol) (PVA) gel electrolyte owing to their mechanical strength and flexibility. According to the discharge curves (Figure 3f), the voltage and capacity are tripled for the serial (blue line) and parallel (red line) assemblies, respectively, thereby increasing the power and energy output. Because of their high energy/power density, the woven BNF yarn batteries can simultaneously power and light up seven blue LEDs and six green LEDs with a dazzling brightness (inset of Figure 3f). 

## 3. Materials and Methods

### 3.1. Chemicals 

For the yarn electrode materials, aerogel multiwalled CNT sheets were drawn from 320 μm high CNT forests (A–Tech System Co., Hwaseong–si, Korea). Regarding the AMs for energy storage, the cathode and anode contained commercially available Ni(OH)_2_ powder with 1 μm particle diameter and Fe_3_O_4_ powder with 50–100 nm particle diameter, respectively. 

In addition, 6M KOH aqueous electrolyte was prepared by dissolving 33.6 g KOH in 100 mL deionized water. To prepare the PVA+KOH solid gel electrolyte, 2 g PVA and 6.7 g KOH were mixed in 20 mL deionized water. The PVA + KOH solution was stirred at 130 °C until it was transparent and viscous. 

### 3.2. Preparation Biscrolled Yarn Battery and B–Biscrolled Yarn Battery 

Four 10 mm wide and 75 mm long CNT sheets were stacked onto a glass plate. To add the AMs onto the CNT sheets, their powders were dispersed in ethanol and dropped onto the CNT sheets; subsequently, the samples were dried at room temperature. To convert 2D sheets into flexible 1D yarn, one end of the AM/CNT sheets was attached to the electrical motor and twisted at approximately 1200 turns/m. Afterward, the parallel biscrolled yarn cathode and anode were connected to copper wires with silver paste and coated with epoxy to prevent unwanted electrochemical side reactions. The b–biscrolled yarn was prepared by wrapping the yarn into the CNT buffer layer; it consisted of three layers of 10 mm wide and 75 mm long CNT sheets that had parallelly been drawn on a vernier caliper (Mitutoyo, Japan). The prepared biscrolled yarn was placed on it. In the next step, the sample was rolled into a cylindrical cone. This cone was densified by inserting twists (approximately 500 turns) and dropping ethanol onto the yarn.

### 3.3. Characterization 

The morphological information was obtained with SEM (S–4600, Hitachi, Japan) and optical microscopy (D750, Nikon, Japan). In addition, an electrochemical analyzer (Vertex EIS, Ivium) was used for all the electrochemical measurements.

### 3.4. Electrochemical Calculation 

The weight of the AM wt% was calculated as follows:

$$wt\%=\frac{W_{total}-W_{CNT}}{W_{total}}\times 100=\frac{W_{AM}}{W_{total}}\times 100$$

where wt% is the weight percentage of the AM NPs, $W_{total}$ is the total weight of the biscrolled yarn battery, $W_{CNT}$ is the weight of neat CNT yarn, and $W_{AM}$ is the weight of the AM NPs. The loading density was calculated by normalizing $W_{AM}$ to the volume of the biscrolled yarn.

The capacity was calculated as follows:

$$Capacity=\frac{I_{discharge}-\Delta t}{Unit}$$

where $I_{discharge}$ is the discharge current, Δt is the discharge time, and Unit is the length, volume, area, and mass of the yarn battery.

## 4. Conclusions

We built high–performance aqueous Ni–Fe yarn batteries by embedding AMs into the inner structure of helical CNT yarns. The employed biscrolling technique enabled the high loading of AMs onto the cathode (Ni: up to 98.5 wt%) and anode (Fe: up to 97.4 wt%). These highly loaded BNF yarn–based batteries delivered a maximum energy density of 422 mWh/cm^3^ and a power density of 7535 mW/cm^3^; thereby, they perform better than most previously reported Ni–Fe batteries. More importantly, owing to the introduction of external CNT buffer layers, the b–BNF yarn battery can retain up to 60.1% of its initial large linear capacity (0.053 mAh/cm) even after 300 charge/discharge cycles with almost 100% coulombic efficiency. Therefore, the presented high–performance b–BNF yarn battery is a promising next–generation energy source for wearable and portable electronics.

## Data Availability

Not applicable.

## Supplementary Materials

The following supporting information can be downloaded at: https://www.mdpi.com/article/10.3390/ijms24021067/s1.
