# Supplementary material for: High–Performance Biscrolled Ni–Fe Yarn Battery with Outer Buffer Layer
Source: Int J Mol Sci. 2023 Jan 5;24(2):1067. doi: 10.3390/ijms24021067 (PMC9864127; doi:10.3390/ijms24021067)
Supplement: Supplementary file 1 [file ijms-24-01067-s001.zip › ijms-2134813-supplementary.pdf]

Supplementary Information

High–Performance Biscrolled Ni–Fe Yarn Battery with Outer Buffer Layer

Jin Hyeong Choi [1,†], Juwan Kim [1,†], Jun Ho Noh [1, 2], Gyuyoung Lee [1], Chaewon Yoon [1], Ui Chan Kim [1], In Hyeok Jang [1], Hae Yong Kim [1] and Changsoon Choi [1, 3,*]

[1] *Department of Energy and Materials Engineering, Dongguk University, 30 Pildong¬ro, 1-gil, Jung¬gu, Seoul 04620, Republic of Korea*

[2] *Department of Advanced Battery Convergence Engineering, Dongguk University, 30 Pildong¬ro, 1¬gil, Jung-gu, Seoul 04620, Republic of Korea*

[3] *Research Center, Sillo Incorporation, 30 Pildong¬ro, 1¬gil, Jung¬gu, Seoul 04620, Republic of Korea*

***Corresponding author: cschoi84@dongguk.edu (C. Choi)**

[†] These authors contributed equally to this work.

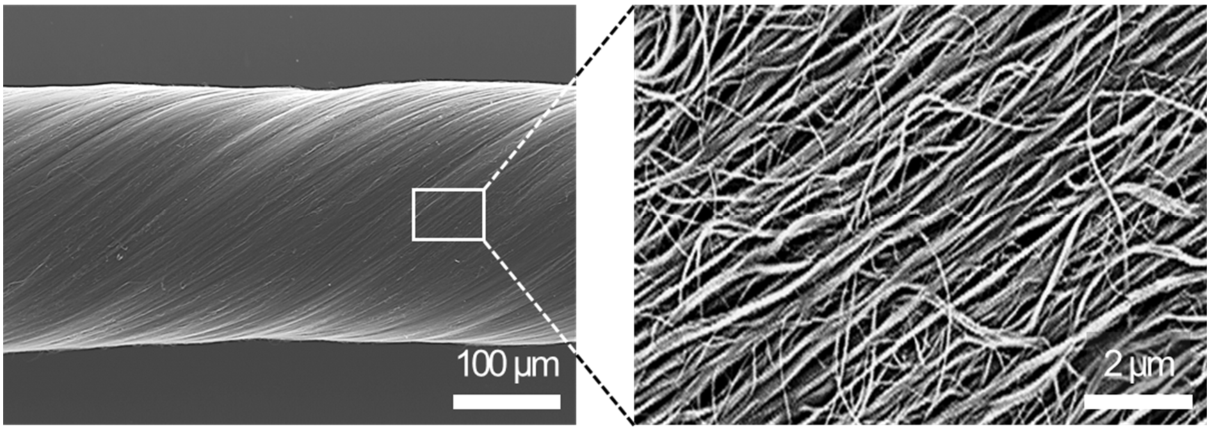

Figure S1. SEM images of neat CNT yarn with magnified area of highly oriented CNT bundles with porous structures.

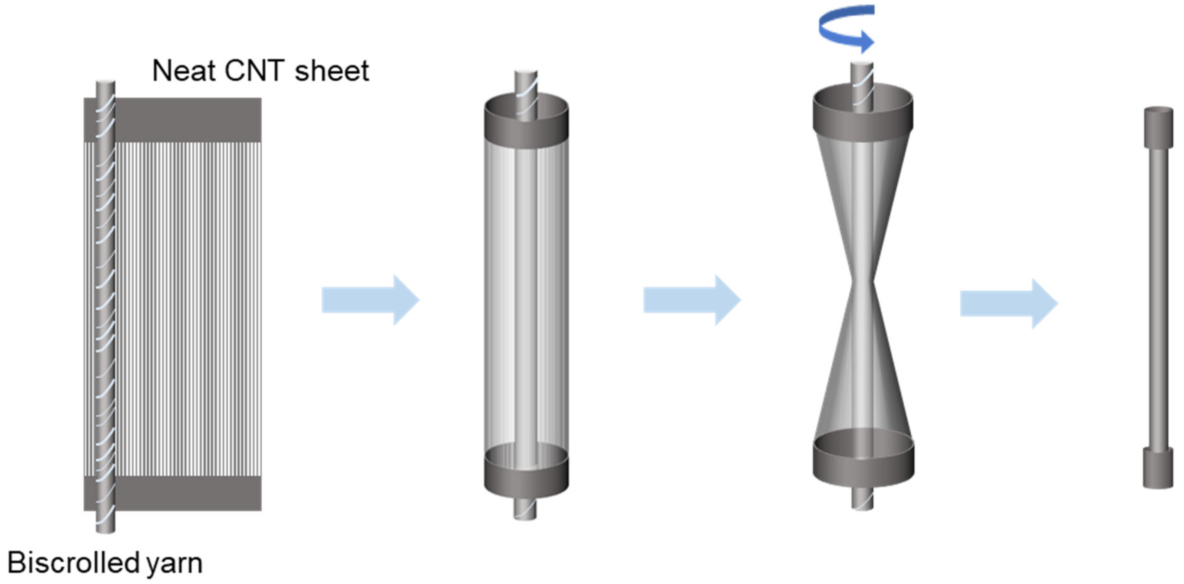

Figure S2. Schematics of wrapping process of neat CNT sheets as buffer layers onto biscrolled yarn.

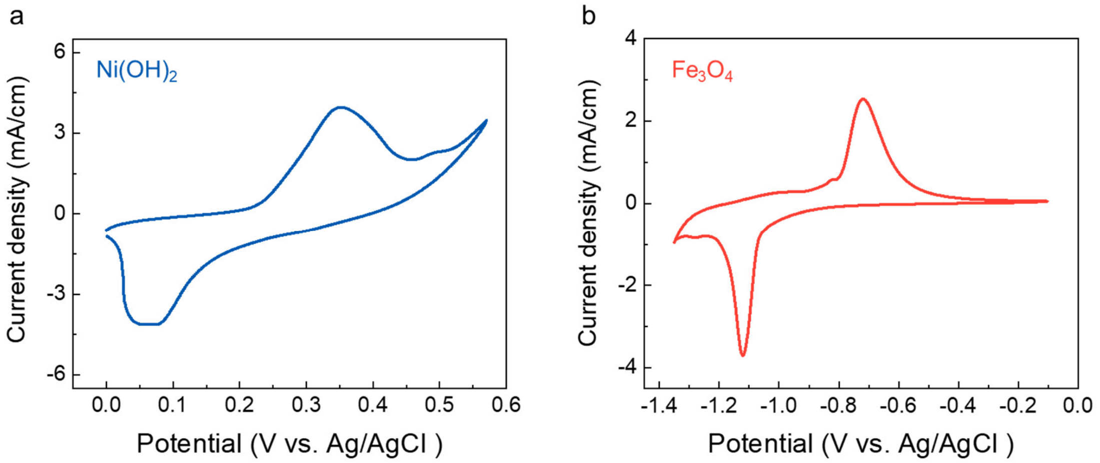

Figure S3. CV curves of (a) $Ni(OH)_2$/CNT biscrolled yarn and (b) Fe_3O_4/CNT biscrolled yarn at 5 mV/s scan rate.

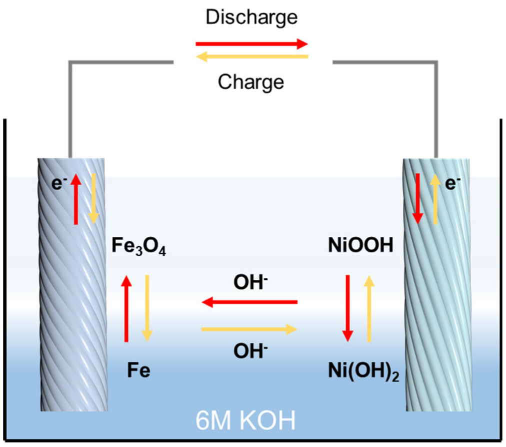

Figure S4. Working principle of Ni-Fe battery and involved electrochemical reactions.

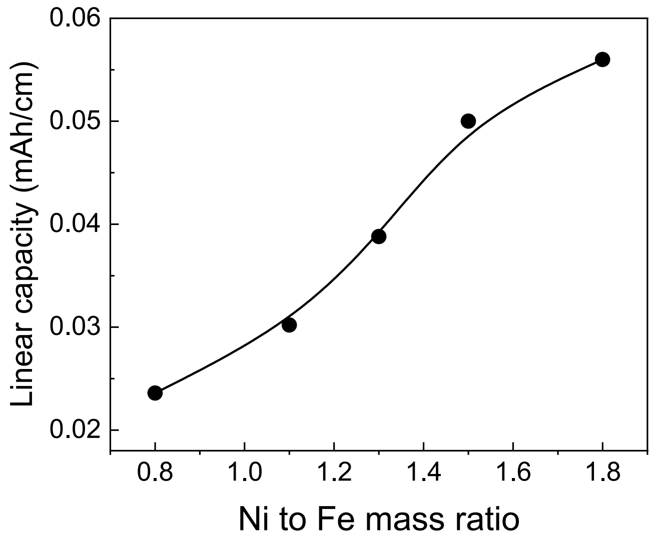

Figure S5. Experimentally derived linear capacity as a function of Ni–to–Fe mass ratio (amount of Fe in the anode is constant).

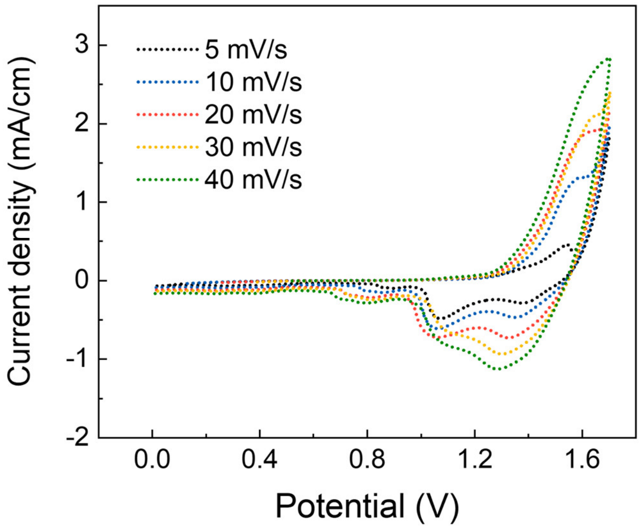

Figure S6. CV curves of BNF yarn battery in aqueous 6M KOH at different scan rates.

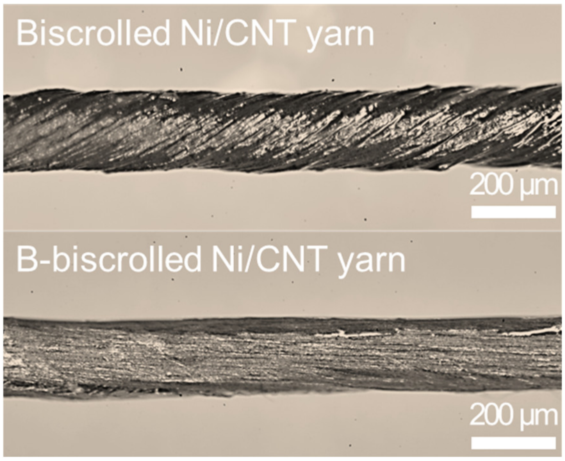

Figure S7. Optical images showing biscrolled Ni/CNT yarn (top) and b-biscrolled Ni/CNT yarn (bottom) after charge/discharge cycles.
